# Sapovirus Translation Requires an Interaction between VPg and the Cap Binding Protein eIF4E

**DOI:** 10.1128/JVI.01650-14

**Published:** 2014-11

**Authors:** Myra Hosmillo, Yasmin Chaudhry, Deok-Song Kim, Ian Goodfellow, Kyoung-Oh Cho

**Affiliations:** a Laboratory of Veterinary Pathology, College of Veterinary Medicine, Chonnam National University, Gwangju, Republic of Korea; b Division of Virology, Department of Pathology, University of Cambridge, Addenbrooke's Hospital, Cambridge, United Kingdom

## Abstract

Sapoviruses of the Caliciviridae family of small RNA viruses are emerging pathogens that cause gastroenteritis in humans and animals. Molecular studies on human sapovirus have been hampered due to the lack of a cell culture system. In contrast, porcine sapovirus (PSaV) can be grown in cell culture, making it a suitable model for understanding the infectious cycle of sapoviruses and the related enteric caliciviruses. Caliciviruses are known to use a novel mechanism of protein synthesis that relies on the interaction of cellular translation initiation factors with the virus genome-encoded viral protein genome (VPg) protein, which is covalently linked to the 5′ end of the viral genome. Using PSaV as a representative member of the Sapovirus genus, we characterized the role of the viral VPg protein in sapovirus translation. As observed for other caliciviruses, the PSaV genome was found to be covalently linked to VPg, and this linkage was required for the translation and the infectivity of viral RNA. The PSaV VPg protein was associated with the 4F subunit of the eukaryotic translation initiation factor (eIF4F) complex in infected cells and bound directly to the eIF4E protein. As has been previously demonstrated for feline calicivirus, a member of the Vesivirus genus, PSaV translation required eIF4E and the interaction between eIF4E and eIF4G. Overall, our study provides new insights into the novel mechanism of sapovirus translation, suggesting that sapovirus VPg can hijack the cellular translation initiation mechanism by recruiting the eIF4F complex through a direct eIF4E interaction.

**IMPORTANCE** Sapoviruses, which are members of the Caliciviridae family, are one of the causative agents of viral gastroenteritis in humans. However, human sapovirus remains noncultivable in cell culture, hampering the ability to characterize the virus infectious cycle. Here, we show that the VPg protein from porcine sapovirus, the only cultivatable sapovirus, is essential for viral translation and functions via a direct interaction with the cellular translation initiation factor eIF4E. This work provides new insights into the novel protein-primed mechanism of calicivirus VPg-dependent translation initiation.

## INTRODUCTION

Sapoviruses belong to the Caliciviridae family and are recognized to be a cause of acute gastroenteritis worldwide (1–3). On the basis of the complete capsid sequence, sapoviruses are divided into five genogroups (genogroup I [GI] to GV). Genogroups I, II, IV, and V are known to infect humans, whereas porcine sapovirus (PSaV) belongs to genogroup III. An increasing prevalence of sapovirus infections has been described, highlighting the emerging role of sapoviruses as a public health problem (2, 4). Despite this, limited studies on the molecular mechanisms of the sapovirus infectious cycle have been conducted due to the lack of a cell culture system. PSaV, the prototype of the genus, has a permissive culture system (5, 6), making it a suitable model for understanding the infectious cycle and molecular mechanisms of sapovirus translation and replication.

Sapoviruses have a small, single-strand, positive-sense RNA genome of approximately 7.3 to 7.5 kb that is predicted to contain two or three open reading frames (ORFs) (7, 8). ORF1 encodes 7 nonstructural (NS) proteins and the major capsid protein (VP1). The NS5 region encodes the viral protein genome (VPg) protein, which in other caliciviruses has been found to be covalently linked to the 5′ terminus of murine norovirus (MNV) and feline calicivirus (FCV) RNA (9, 10). PSaV ORF2, the equivalent of FCV ORF3, is thought to encode a minor structural protein that, by analogy with other caliciviruses, may also play a role in viral replication (11, 12). Sapovirus ORF3 is present in some strains of the virus, but its function is as yet unknown (7).

As with all positive-sense RNA viruses, sapovirus translation initiates immediately upon cell entry, with the viral genome acting as an mRNA template. The expression of viral proteins is frequently subject to regulation at the level of the initiation of mRNA translation. There are at least 12 eukaryotic translation initiation factors (eIFs) recruited during the initial stage of protein synthesis (13). Among these, a cap binding complex (eIF4F) binds to the 5′ end of the cellular mRNAs and recruits other factors to form a highly stable ribonucleoprotein complex for protein synthesis (13, 14). eIF4F is a heterotrimeric complex consisting of cap binding protein eIF4E, scaffold protein eIF4G, and an RNA helicase (eIF4A). During the translation initiation process, eIF4E binds to the 7-methylguanosine (m^7^-G) cap structure of the host mRNA and then recruits and activates eIF4G and eIF4A. eIF4A unwinds the mRNA at the 5′ end and facilitates ribosome binding (13). eIF4G acts as the cornerstone for the multisubunit eIF4F complex, linking the mRNA cap and ribosomal subunit via eIF4E and eIF3 binding domains, respectively (13).

Many RNA viruses have developed unique mechanisms to usurp the host cell translation machinery for viral protein synthesis. Cap-independent translation is well characterized in the Picornaviridae, where internal ribosomal entry site (IRES)-mediated translation is used (15). Recently, another cap-independent translation mechanism primed by a virus genome-encoded protein has been characterized in caliciviruses. Like picornavirus, calicivirus genomes do not possess a 5′ cap structure; instead, a VPg is covalently linked to the 5′ end of the genome (16, 17). Recent studies have demonstrated a critical role for VPg in calicivirus protein synthesis (9), as treatment of FCV or MNV VPg-linked RNA with proteinase K (Pk) abrogated translation and infectivity (9, 10). The Norwalk virus VPg protein also interacts with eIF3 (18, 19). FCV VPg-dependent translation is directly coupled with the interaction of VPg with the cap binding protein eIF4E, and eIF4E is functionally required for FCV translation (9). MNV translation is not affected by depletion of eIF4E or by the separation of the eIF4E binding domain from eIF4G but shows a functional requirement for eIF4A (9). This suggests that within the Caliciviridae family, the functional requirements for eIF4F components differ (9, 20–22).

The mechanism of translation initiation used by sapoviruses is currently unknown. Therefore, to characterize the role of sapovirus VPg in initiation of viral protein synthesis, we performed a series of biochemical and *in vitro* studies. We have demonstrated that the linkage of VPg to the PSaV genome is required for the translation and infectivity of viral RNA. We also show a clear role for a VPg-eIF4E interaction in PSaV translation, indicating that PSaV is functionally similar to FCV. These results contribute to a more detailed understanding of sapovirus translation and the role of the PSaV VPg protein during initiation of viral protein synthesis.

## MATERIALS AND METHODS

### Virus, cells, and reagents.

The PSaV Cowden strain was obtained from K. O. Chang (Kansas State University) and is a tissue culture-adapted strain recovered from the full-length infectious clone pCV4A (23). PSaV-permissive LLC-PK cells were transduced with lentiviruses expressing the bovine viral diarrhea disease virus (BVDV) Npro protein to produce an interferon (IFN)-deficient cell line, which allows more efficient virus replication (M. Hosmillo, F. Soorgeloos, R. Hiraide, I. Goodfellow, and K.-O. Cho, submitted for publication). IFN-deficient LLC-PK cell-derived transduced cells expressing BVDV NPro (LLC-PK-Npro cells) were used to propagate PSaV in Eagle's minimal essential medium (EMEM) supplemented with 200 μM glycochenodeoxycholic acid (GCDCA; Sigma), 2.5% fetal calf serum, and 1% penicillin-streptomycin (P/S) at 37°C with 5% CO_2_.

Antisera to PSaV VPg and capsid were generated by immunization of New Zealand White rabbits with purified recombinant VPg and virus, respectively. Antisera against eIF4E, eIF4A, and eIF4G1 were purchased from Cell Signaling Technology.

The cap analogue (m^7^-G-5′-ppp-5′-G; Promega), elastatinal (Calbiochem), proteinase K (Ambion), and [^35^S]methionine (PerkinElmer) were purchased. Recombinant eIF4E binding protein (4E-BP1) was a kind gift from Simon Morley (University of Sussex), and the recombinant foot-and-mouth disease virus (FMDV) Lb protease was provided by Tim Skern (University of Vienna). The eIF4E expression plasmid was kindly received from Stephen Curry (Imperial College London). Recombinant eIF4E was first purified with a His tag by affinity chromatography on a HiTrap chelating column (GE Healthcare). To ensure a functional eIF4E protein, purified eIF4E was then additionally purified via an m^7^-GTP Sepharose column (GE Healthcare) using a salt gradient elution to avoid possible contamination with a cap analogue (9).

### Expression and purification of recombinant PSaV VPg.

The cDNA encoding PSaV VPg was PCR amplified from a full-length clone, pCV4A, using primers VPg-F (5′-GCG ACC ATG GCG AAA GGG AAA AAC AAA CGC) and VPg-R (5′-TTA CTC GAG TCA CTC ACT GTC ATA GGT GTC ACC). PCR amplicons were cloned into pProEX HTc digested with NcoI and XhoI (underlined in the sequences presented above), and the resulting constructs were verified by sequencing.

The VPg-containing plasmid was expressed in the B834(DE3) Escherichia coli strain. Large-scale recombinant protein production was performed in autoinducible ZYP medium (1% N-Z-Amine, 0.5% yeast extract, and salts) containing antibiotics, and cells were cultured for 2 days at 37°C with shaking. Cell pellets from the bacterial culture were resuspended in a lysis buffer containing 20 mM Tris-HCl, pH 7.5, 500 mM NaCl, and one tablet of EDTA-free protease inhibitor (Roche). Following resuspension, the pellets were digested and sonicated. The lysates were collected and loaded onto a 5-ml HisTrap HP column (GE Healthcare), and His-tagged VPg proteins were eluted in 500 mM imidazole. To prepare the untagged VPg protein, the His tag from VPg was digested by tobacco etch virus protease (kindly provided by Jeong-sun Kim, Chonnam National University) at 18°C overnight and purified again on the HisTrap HP column. The final recombinant untagged VPg protein was concentrated and snap-frozen in liquid nitrogen before storage at −80°C.

### VPg-dependent *in vitro* translation.

LLC-PK-Npro cells were mock infected or infected with PSaV at a multiplicity of infection (MOI) of 10 50% tissue culture infective doses (TCID_50_s)/cell. At 12, 24, and 48 h postinfection, the cells were harvested and total RNA was extracted using a GenElute total RNA extraction kit (Sigma). *In vitro* translation reactions using Flexi rabbit reticulocyte lysate (RRL; Promega) were performed as previously described (9) using 40 μg ml^−1^ RNA from mock- or PSaV-infected cells and 12.5 μg ml^−1^ *in vitro*-transcribed control RNAs. Capped control dicistronic mRNAs containing either FMDV or porcine teschovirus 1 (PTV) IRES were synthesized *in vitro* from plasmid pGEM-Rluc/FMDV/Fluc (where Rluc and Fluc represent Renilla and firefly luciferase, respectively) and/or pGEM-CAT/PTV/LUC, provided by Graham Belsham (Technical University of Denmark), respectively (24). Control capped PSaV RNA was *in vitro* transcribed from pCV4A containing the full-length PSaV genome. Transcribed RNAs were then capped using a ScriptCap m^7^-G capping system (Epicentre Biotechnologies).

In reactions that required the addition of cap analogue, 4E-BP1, Lb protease, and eIF4E, recombinant proteins were preincubated at 30°C for 15 min prior to the addition of RNA. Complementation with recombinant eIF4E after eIF4E sequestration or depletion was carried out for an additional 5 min before incubation of the RNA template. After the incorporation of RNA, *in vitro* translation was performed at 30°C for 90 min and terminated with an equal volume of Tris buffer containing 10 mM EDTA and 100 ng ml^−1^ RNase A (Ambion). Translated proteins were resuspended in 5× SDS sample buffer and resolved on 12.5% polyacrylamide gels. Pretreatment of RNAs was carried out by incubation of RNA in 10 mM Tris, pH 8.0, 1.0 mM EDTA, 0.1 M NaCl, and 0.5% SDS in the presence or absence of 10 μg ml^−1^ proteinase K for 30 min at 37°C. Pretreated RNAs were immediately purified by use of the GenElute RNA cleanup protocol (Sigma). Protein synthesis levels were quantitated from dried gels using a Packard instant imager (Canberra, Packard, United Kingdom). X-ray films were developed after 4 to 24 h of incubation at room temperature.

### Immunoprecipitation.

Products from the *in vitro* translation reaction mixtures were incubated with protein A agarose beads and antibodies against MNV NS7 or PSaV VPg or VP1 in radioimmunoprecipitation assay (RIPA) buffer (50 mM Tris, pH 8.0, 150 mM NaCl, 1% NP-40, 0.5% sodium deoxycholate, 0.1% SDS). The mixture was centrifuged and washed with RIPA buffer 3 times, and the immunoprecipitated proteins were then evaluated by autoradiography.

### Avicel-based plaque assay.

Total RNA extracts (10 μg) from mock- or PSaV-infected cells were pretreated with and without proteinase K as described above. Treated or mock-treated RNA was transfected into LLC-PK-Npro cells using the Lipofectamine 2000 reagent per the manufacturer's instructions (Invitrogen). After 4 h, the cells were washed and overlaid with 1.3% Avicel cellulose (FMC Health and Nutrition) in EMEM supplemented with 2.5% fetal bovine serum (FBS), 0.225% sodium bicarbonate, and 1.0% penicillin-streptomycin. The plates were incubated at 37°C for 4 days. After incubation, the Avicel mixture was removed and the cells were fixed and stained with 1.6% methylene blue and with 33.3% formaldehyde solution in 1× phosphate-buffered saline for 30 min. Plates were washed with distilled water until the desired staining intensity was achieved. The infectious virus titer was quantified by the plaque assay method.

### Cap-Sepharose purification for eIF4F complex.

Cell lysates were prepared from either mock- or PSaV-infected (MOI, 10 TCID_50_s/cell) LLC-PK-Npro cells in cap-Sepharose lysis buffer (100 mM KCl, 0.1 mM EDTA, 10% glycerol, 2 mM MgCl_2_, 20 mM HEPES, pH 7.6) and RNase treated for 15 min at room temperature. Cytoplasmic extracts were centrifuged and incubated with m^7^-GTP Sepharose (GE Healthcare) overnight at 4°C. The eIF4F-enriched complex was precipitated and washed 2 times with lysis buffer. Bound proteins were eluted in 2× reducing buffer, resolved by SDS-PAGE, and analyzed by immunoblotting.

### ELISA-based initiation factor capture assay.

An enzyme-linked immunosorbent assay (ELISA)-based capture assay was performed as described previously (25). Briefly, purified PSaV VPg (1 μg) was coated onto 96-well plates (Nunc Immunosorb) overnight at 4°C. The wells were then blocked with 2% bovine serum albumin (BSA) in Tris-buffered saline (TBS). HeLa cell lysates or recombinant eIF4E was diluted in 2% BSA in TBS with 0.05% Tween 20, and the mixture was incubated for 2 h at 4°C in plates whose wells had previously been coated. The association of eIF4E, eIF4A, and eIF4G was detected using specific primary antibodies. Secondary horseradish peroxidase (HRP)-conjugated antibodies were added, and signals were developed with tetramethylbenzidine substrate. The reaction was stopped with H_2_SO_4_, and the optical density at 450 nm was determined.

### Pulldown assay.

His-tagged PSaV VPg was immobilized on HisPur cobalt resin (Pierce) and incubated with either recombinant eIF4E or RNase-treated cell lysates on the rotator for 4 h and overnight, respectively, at 4°C. Proteins interacting with His-tagged VPg were pulled down by centrifugation and sequentially washed 3 to 5 times in lysis buffer. Bound proteins were eluted with 400 mM imidazole. Binding to eIF4F components or recombinant eIF4E was analyzed by Western blotting.

### eIF4E siRNA-based functional assay.

Confluent LLC-PK-Npro cells were transfected with either small interfering RNA (siRNA) against a nonspecific target (100 pmol) or eIF4E (100 pmol) using the Lipofectamine 2000 reagent following the manufacturer's instructions. Cells were then infected at an MOI of 1 TCID_50_/cell. Unabsorbed viruses were removed after 4 h, and infected cells were maintained in EMEM with 2.5% FBS and 200 μM GCDCA. The virus titer was analyzed at 24 h postinfection by quantitative reverse transcription-PCR using PSaV-specific primers targeting viral protease (forward primer 5′-CAACAATGGCACAACAACG-3′ and reverse primer 5′-ACAAGCTTCTTCACCCCACA-3′).

## RESULTS

### Porcine sapovirus RNA is linked to VPg.

**FIG 1:**
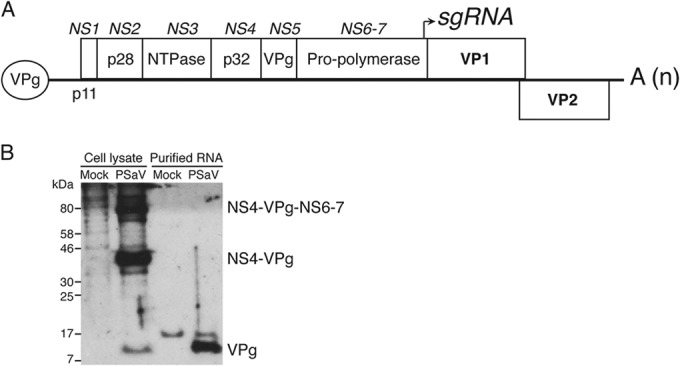
The porcine sapovirus genome is linked to the fully processed VPg at its 5′ terminus. (A) A schematic presentation of the porcine sapovirus genome showing the nonstructural (NS1 to NS6-7) and structural (VP1 and VP2) proteins. sgRNA, subgenomic RNA. A, poly(A) tail in different lengths (n). (B) Monolayers of LLC-PK cells were either mock infected or infected with PSaV at an MOI of 10 TCID_50_s/cell. After adsorption, the medium was replaced with EMEM containing GCDCA and FBS. Infection proceeded for 30 h, and then cell lysates and purified RNA were prepared. Purified RNAs were subjected to RNase treatment, and then the cell lysates and treated RNA were subsequently analyzed by Western blotting.

On the basis of data obtained with other genera of the Caliciviridae, sapovirus RNA is predicted to be VPg linked (Fig. 1A). To date, however, no experimental evidence for the formation of VPg-linked sapovirus RNA has been described. To determine if VPg is linked to the PSaV genome, we examined lysates and purified RNAs from infected LLC-PK-Npro cells for the presence of VPg by Western blotting. It is important to note that we used mock- or PSaV-infected interferon-deficient LLC-PK-Npro cells for total RNA extractions to enable us to harvest a sufficient amount of viral RNA for the *in vitro* translation reactions. Using this cell line, our previous studies showed a 100-fold increase in virus replication, and thus, these cells were consequently used for virus propagation (Hosmillo et al., submitted). This cell line is referred to as LLC-PK-Npro throughout the report.

Cell lysates harvested from PSaV-infected cells showed the presence of a fully processed form of VPg (15 kDa) and two VPg-containing precursors from polyprotein which, on the basis of their molecular masses, corresponded to NS4-VPg (46 kDa) and NS4-VPg-NS6-7 (85 kDa) (Fig. 1B). To determine which form of VPg is linked to the viral genome, we examined purified RNA isolated from mock- and PSaV-infected cells for the presence of VPg after RNase treatment. Western blot analysis revealed that only the fully processed form of VPg is linked to the PSaV genome (Fig. 1B), as has been observed for MNV (9). This confirms that, like the other genera of the Caliciviridae, the genome of PSaV, a member of the Sapovirus genus, is also covalently linked at the 5′ end.

### Porcine sapovirus VPg is required for infectivity and the translation of viral RNA.

**FIG 2:**
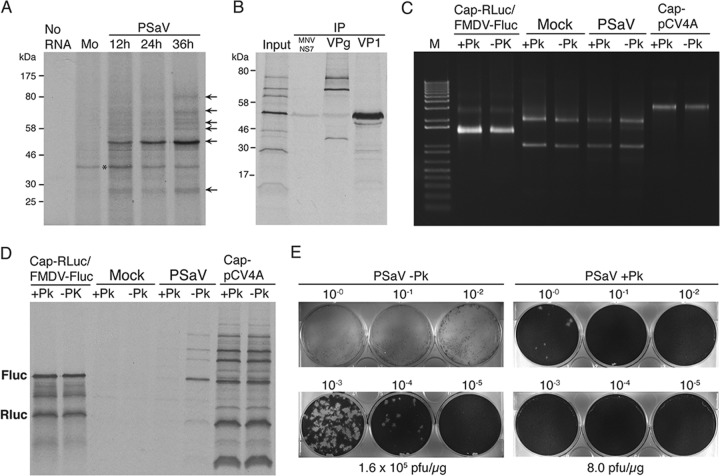
Porcine sapovirus requires VPg linkage to confer virus infectivity and viral RNA translation. (A) Monolayers of LLC-PK cells were either mock infected or PSaV infected at an MOI of 10 TCID_50_s/cell. Cells were harvested at 12, 24, and 36 h postinfection and lysed, and the total RNA was extracted. RNA isolated from infected cells during the time course was used to program the *in vitro* translation reaction using RRLs. The translation products were analyzed by SDS-PAGE, and the translation profiles were then evaluated by autoradiography. An asterisk highlights the position of a cellular protein that is translated in a cap-dependent mechanism from a highly abundant cellular mRNA present in the RNA preparations. Lane Mo, RNA from mock-infected cells. (B) Proteins produced by *in vitro* translation were immunoprecipitated (IP) overnight using antibodies against the murine norovirus NS7 protein, PSaV VPg, and capsid proteins. Immunoprecipitates were washed 3 times with RIPA buffer and then resolved by 12.5% SDS-PAGE. Protein precipitates were analyzed by autoradiography. (C) RNA samples prepared from *in vitro*-transcribed and capped cap-Rluc-FMDV IRES-Fluc dicistronic RNA (Cap-Rluc/FMDV-Fluc), mock- and PSaV-infected cells, and capped *in vitro* transcripts from the PSaV full-length cDNA clone pCV4A (Cap-pCV4A) were pretreated with or without Pk at 37°C for 30 min. Following RNA purification, the purified RNA samples were analyzed by nondenaturing agarose gel electrophoresis to confirm their integrity. (D) RNA samples were then used to program RRLs, and the RNA was subjected to an *in vitro* translation reaction. (E) RNA extracted from mock- or proteinase K-treated, PSaV-infected cells was transfected into LLC-PK cells expressing BVDV Npro. Serial 10-fold dilutions of the RNA preparations were transfected, and at 4 days posttransfection the cells were washed and incubated in 1.3% Avicel-based overlay medium containing 2.5% FBS and 0.225% sodium bicarbonate and supplemented with 200 μM GCDCA. Cells were immediately fixed and stained.

To characterize the translation initiation of PSaV, RRLs were used to examine the ability of the VPg-linked RNA to translate *in vitro*. Total RNA was extracted from either mock- or PSaV-infected cells and used to program RRLs for *in vitro* translation reactions. The profile of translation from PSaV RNA showed several additional proteins compared with the number of proteins from RNA from mock-infected cells (Fig. 2A). These additional proteins were predicted to be of viral origin, as the amount of viral RNA used to produce these proteins increased during the course of infection. To determine if the additional protein products were, in fact, PSaV specific, immunoprecipitations were performed using antisera specific to several PSaV proteins. Using antibodies against the PSaV VPg protein and capsid protein (VP1), proteins with molecular masses corresponding to the predicted molecular masses of VP1 and the precursor forms of VPg were immunoprecipitated (Fig. 2B). As expected, no significant levels of PSaV proteins were immunoprecipitated with antiserum to the MNV NS7 protein, although a small amount of the capsid protein was often detected due to the high levels of expression (Fig. 2B).

To determine if PSaV translation is VPg dependent, we examined the effect of proteinase K (Pk) treatment on the translation profile of RNA. As controls, we used RNAs isolated from mock-infected cells and capped *in vitro*-transcribed RNA of a dicistronic construct expressing the chloramphenicol acetyltransferase (CAT) protein in a cap-dependent manner and the Fluc protein dependent on the FMDV IRES structure. In addition, we used capped *in vitro*-transcribed full-length PSaV RNA. Following Pk treatment, RNA samples were confirmed to be intact before they were subjected to *in vitro* translation and transfection (Fig. 2C). The translation of VPg-linked PSaV RNA was abrogated by prior treatment with Pk (Fig. 2D), confirming that VPg linkage is necessary for viral translation *in vitro*. To further confirm the role of VPg in PSaV translation during replication in cells, we then examined the effect of Pk treatment on PSaV RNA infectivity. We measured the specific infectivity of Pk- or mock-treated PSaV RNA in permissive LLC-PK cells. Without Pk pretreatment, PSaV RNA yielded up 1.6 × 10^5^ PFU/μg of total RNA, but this was markedly reduced to 8 PFU/μg after Pk treatment (Fig. 2E).

### Porcine sapovirus VPg is associated with cellular translation initiation factors during virus infection.

**FIG 3:**
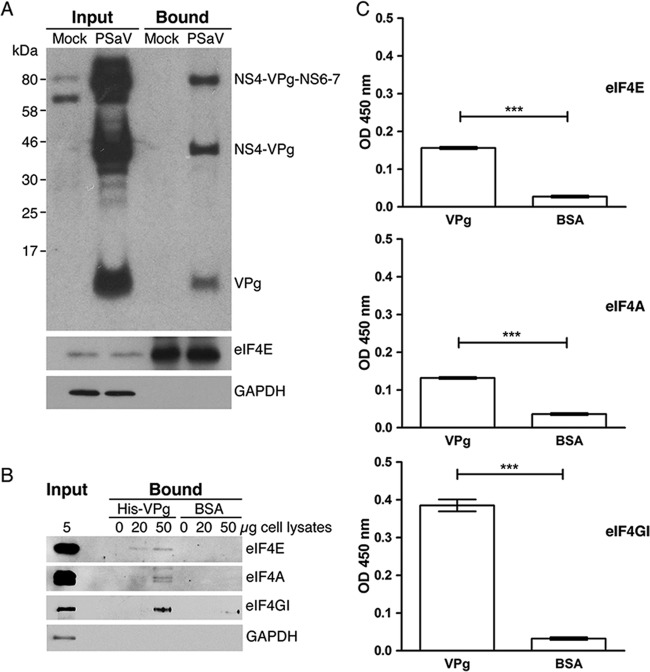
The porcine sapovirus VPg binds to the cellular translation initiation factors during virus infection. (A) Monolayers of LLC-PK cells transduced with BVDV Npro were either mock infected or infected with PSaV at an MOI of 10 TCID_50_s/cell. At 36 h postinfection, cells were collected and lysed in cap-Sepharose buffer. Lysates were centrifuged and further treated with RNase. One thousand micrograms of lysates was incubated with cap-Sepharose beads overnight. After they were washed, the bound proteins were analyzed by SDS-PAGE and Western blotting. (B) A His tag pulldown assay was performed using 10 μg of His-tagged VPg or BSA immobilized on HisPur cobalt resin. Increasing amounts (0 to 50 μg) of nuclease-treated cell lysates were then incubated overnight with the bait VPg protein. Protein complexes were extensively washed, eluted, and analyzed by Western blotting. eIF4GI, eIF4 gamma 1. (C) A capture ELISA was performed using 1 μg of purified recombinant PSaV VPg or BSA as a control. Ten micrograms of nuclease-treated cytoplasmic extracts was incubated with either target, and the extracts were extensively washed prior to detection with rabbit antibodies to eIF4E, eIF4A, or eIF4G. Antibody binding was detected using a secondary antirabbit HRP-conjugated antibody, followed by incubation with ELISA substrate. Samples were analyzed in triplicate and in at least three independent experiments. One representative set of data is shown. Error bars represent standard deviations for triplicate samples. OD, optical density.

Previous studies on FCV and MNV translation revealed the association of VPg with cellular translation initiation factors during viral replication in cell culture (9, 10). Given our observation that PSaV VPg was required for viral translation and infectivity, this also suggested that PSaV VPg is associated with the cellular translation machinery. To investigate if the PSaV VPg protein interacted with the translation initiation factor (eIF4F) complex, we purified the eIF4F complex from mock- and virus-infected cells using m^7^-GTP Sepharose resin, which binds to eIF4E and therefore can be used to enrich eIF4F-interacting proteins. The eIF4E-containing complex isolated from infected cells also contained PSaV VPg as both precursor and fully processed forms (Fig. 3A). As expected, the eIF4E protein was isolated by m^7^-GTP Sepharose, whereas GAPDH (glyceraldehyde-3-phosphate dehydrogenase) was not, confirming the specificity of the assay (Fig. 3A). To examine the interaction of PSaV VPg and the eIF4F complex from the cell lysates, immobilized recombinant His-tagged VPg protein was incubated with nuclease-treated cell lysates, and associated cellular factors were detected by Western blotting. Western blot analysis showed that the components of eIF4F were pulled down with His-tagged VPg but not with BSA (Fig. 3B). No interaction between VPg and GAPDH was observed, verifying the specificity of the pulldown assay. To further confirm the association of VPg with components of the eIF4F complex, a capture ELISA-based binding assay was performed whereby wells were coated with recombinant PSaV VPg or BSA and then incubated with lysates from a permissive cell. The retention of the eIF4F complex was then examined by ELISA using anti-initiation factor antibodies. VPg retained the eIF4E, eIF4A, and eIF4G components of the eIF4F complex, confirming the association of PSaV VPg with these cellular translation initiation factors (Fig. 3C). As expected, control wells coated with BSA did not show any interaction with the eIF4F components (Fig. 3C).

### Porcine sapovirus VPg binds to recombinant eIF4E directly.

**FIG 4:**
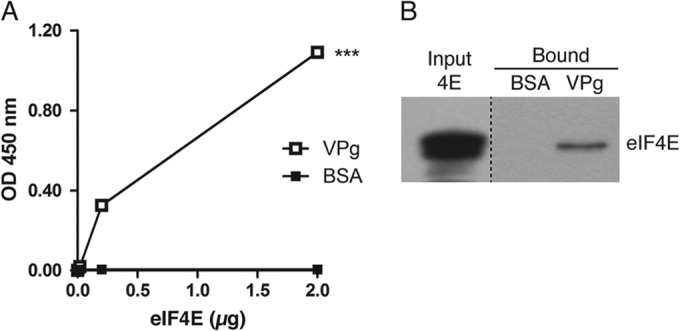
Porcine sapovirus VPg binds to recombinant eIF4E. (A) A capture ELISA was performed using 1 μg of purified recombinant PSaV VPg or BSA as a control. Increasing concentrations of recombinant eIF4E were then incubated with both coated proteins, and eIF4E was extensively washed prior to detection with rabbit antibodies to eIF4E. Antibody binding was detected using a secondary antirabbit HRP-conjugated antibody, followed by incubation with ELISA substrate. Samples were analyzed in triplicate in at least three independent experiments. One representative set of data is shown. (B) A pulldown assay was performed using 1 μg of recombinant His-tagged VPg immobilized on HisPur cobalt resin or resin alone. Resins with and without VPg were washed, blocked with BSA, and incubated with a similar concentration of either BSA or recombinant eIF4E. Binding to eIF4E was detected by immunoblotting with anti-eIF4E antibody. Samples were analyzed in duplicate and in at least three independent experiments, and one representative set of data is shown.

To determine if VPg interacted directly with eIF4E, as has been observed for other caliciviruses, an ELISA-based capture assay was performed. Plates coated with PSaV VPg, but not BSA, showed direct binding with purified recombinant eIF4E in a dose-dependent manner (Fig. 4A). To confirm this interaction, His-tagged PSaV VPg protein was immobilized on cobalt resin and incubated with recombinant eIF4E in a pulldown assay. Western blot analysis further demonstrated a direct interaction between PSaV VPg and eIF4E, but no interaction was found in resin treated with BSA as a control (Fig. 4B).

### Porcine sapovirus translation is insensitive to the cap analogue but requires an eIF4E-eIF4G interaction.

**FIG 5:**
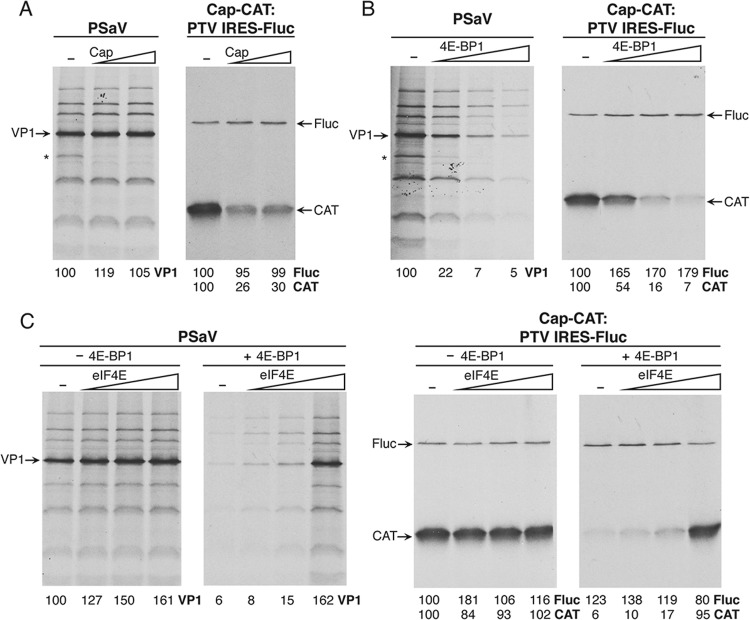
Porcine sapovirus translation is independent of the cap analogue but requires the eIF4E-eIF4G interaction. (A) *In vitro* translation was performed, as illustrated by the experimental scheme above the gels, using either VPg-linked PSaV RNA or dicistronic RNA containing a cap-dependent CAT and PTV IRES-dependent Rluc. Translation reaction mixtures were preincubated with increasing concentrations of CAP analogue, and then the RNAs were added to initiate protein synthesis. The profiles for VPg-, cap-, and IRES-dependent translations were resolved by SDS-PAGE. The gels were fixed, dried, and exposed to X-ray film. The intensity of each band was quantitated with reference to the value obtained in the absence of the cap analogue. (B) *In vitro* translation was performed by following the experimental scheme indicated above the gels and with increasing amounts of recombinant 4E-BP1, before addition of PSaV RNA and dicistronic RNA. RNAs were then added to initiate protein synthesis. The profiles for VPg-, cap-, and IRES-dependent translations were resolved by SDS-PAGE. The gels were fixed, dried, and exposed to X-ray film. The intensity of each band was quantitated with reference samples incubated with 4E-BP1 buffer only. The asterisk highlights the position of a cellular protein that is translated by a cap-dependent mechanism from a highly abundant cellular mRNA present in the RNA preparations. (C) *In vitro* translation was performed as described in the legend to panel B with the addition of recombinant eIF4E after sequestration by 4E-BP1. The effects of separation by 4E-BP1 buffer or 4E-BP1 and subsequent complementation of eIF4E in cap-, VPg-, and IRES-dependent translation were evaluated by SDS-PAGE and autoradiography. In each panel, the numbers beneath the lanes indicate the quantitated protein synthesis in percentage for each corresponding *in vitro* translation reaction.

To further examine the functional requirements of PSaV RNA translation, RRLs were used as an experimental system to probe the relative roles of eIF4E and the eIF4E-eIF4G interaction. To determine whether or not the interaction of eIF4E with the cap analogue inhibits PSaV translation, the effect of increasing concentrations of the cap analogue on PSaV translation was examined. As expected, the cap analogue inhibited cap-dependent translation but had no effect on FMDV IRES-dependent translation (Fig. 5A). In contrast, PSaV translation was unaffected, suggesting that there are distinct sites for VPg and cap binding to eIF4E, fitting with our ability to copurify VPg on m^7^-GTP Sepharose (Fig. 3A). In addition, when eIF4E was sequestered by the addition of recombinant eIF4E binding protein (4E-BP1), translation of PSaV RNA was reduced, as was cap-dependent translation (Fig. 5B). As expected, 4E-BP1 had no effect on PTV IRES-mediated translation (Fig. 5B). The 4E-BP1-mediated inhibition of PSaV- and cap-dependent translation could be restored by the addition of recombinant eIF4E, confirming the specificity of the inhibitory effect of 4E-BP1 on eIF4E (Fig. 5C).

### Porcine sapovirus translation requires an intact eIF4G.

**FIG 6:**
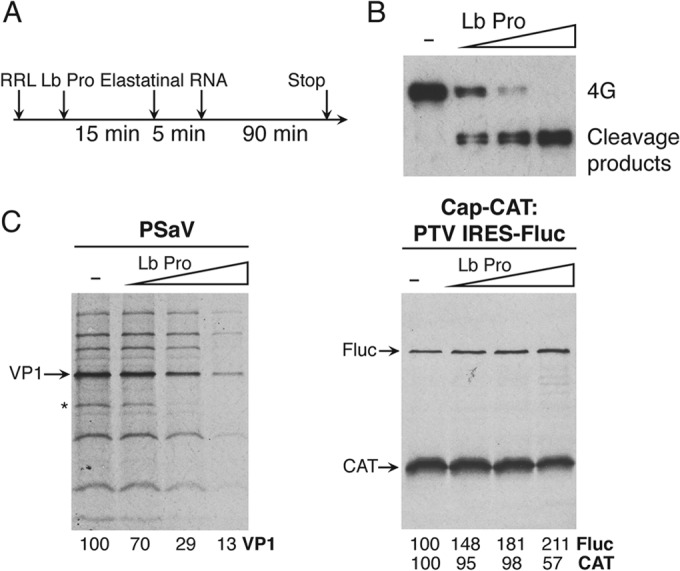
Porcine sapovirus translation requires full-length eIF4G. (A) *In vitro* translation was performed by following the experimental scheme indicated above each gel using increasing amounts of FMDV Lb protease (Lb Pro) to cleave eIF4G. Subsequently, elastatinal was added to quench the protease activity before the addition of PSaV RNA or *in vitro*-transcribed dicistronic RNA. (B) The effect of Lb protease was confirmed by Western blotting using antibody against eIF4G. (C) The profiles of VPg-, cap-, and IRES-dependent translation were resolved by SDS-PAGE. The gels were fixed, dried, and exposed to X-ray film. The intensity of each band was quantitated with reference samples incubated without Lb protease. The asterisk highlights the position of a cellular protein that is translated by a cap-dependent mechanism from a highly abundant cellular mRNA present in the RNA preparations.

To examine the role of the eIF4E-eIF4G interaction and to examine if the N-terminal eIF4E binding domain of eIF4G is required for PSaV translation, RRLs were pretreated with FMDV Lb protease, which cleaves eIF4G, separating the eIF4E binding domain from the eIF4A, PABP, and eIF3 binding domains (Fig. 6A). Cleavage of eIF4G was verified by Western blot analysis, showing increasing eIF4G cleavage with higher concentrations of Lb protease (Fig. 6B). Cleavage of eIF4G reduced the translation of PSaV RNA and cap-dependent translation, whereas PTV IRES-dependent translation was slightly increased (Fig. 6C). A protein that was translated from PSaV RNA preparations and that corresponded to a host mRNA (highlighted with an asterisk in Fig. 6C) was inhibited by eIF4G cleavage.

### Porcine sapovirus translation is sensitive to eIF4E depletion.

**FIG 7:**
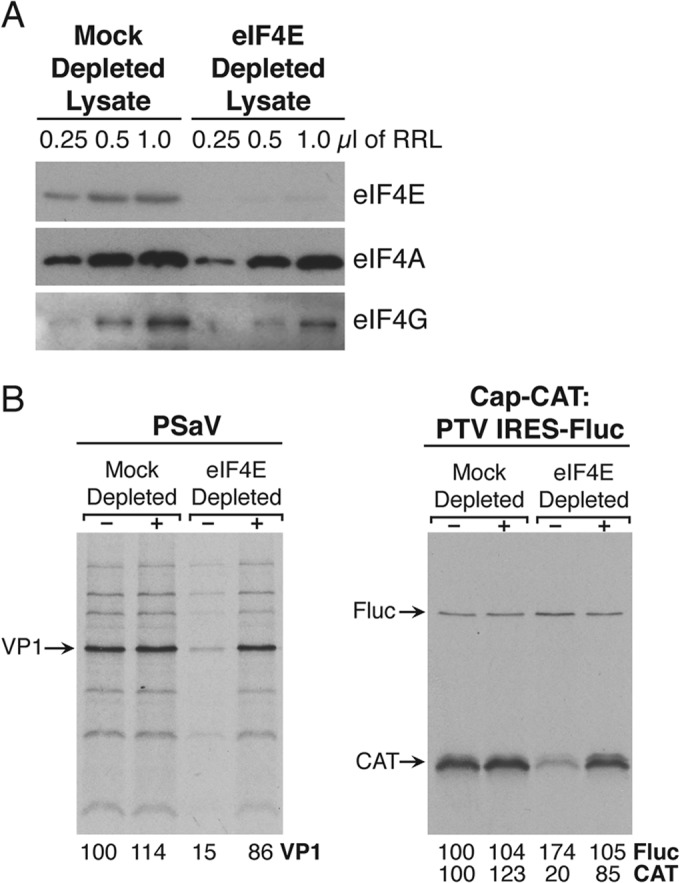
Porcine sapovirus translation is sensitive to eIF4E depletion. Mock-depleted or eIF4E-depleted RRLs were used for the *in vitro* translation reaction in which the reaction mixture was replenished with recombinant eIF4E or buffer alone. (A) Depletion of eIF4E was verified by Western blotting using 0.25, 0.5, and 1.0 μl of the RRLs. (B) The translation of VPg-, cap-, and IRES-dependent proteins was observed in mock- and eIF4E-depleted lysates with and without the addition of recombinant eIF4E.

To further examine the role of the PSaV VPg-eIF4E interaction, the effect of eIF4E depletion on PSaV translation was investigated. RRLs were depleted of eIF4E using m^7^-GTP Sepharose after the addition of 4E-BP1 to prevent the concomitant removal of eIF4G. Depletion of eIF4E was confirmed by Western blot analysis. eIF4E was significantly depleted, whereas the levels of eIF4A and eIF4G remained largely similar to those in mock-depleted lysates (Fig. 7A). Cap- and PSaV-VPg-dependent translation was inhibited by eIF4E depletion; however, PTV IRES-mediated translation was slightly stimulated (Fig. 7B). The addition of recombinant eIF4E restored cap-dependent and PSaV VPg-dependent translations to levels similar to those observed in mock-depleted lysates (Fig. 7B).

### Porcine sapovirus requires eIF4E in cell culture.

**FIG 8:**
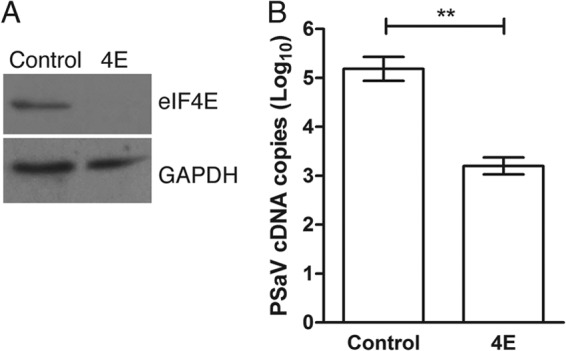
eIF4E is required for PSaV replication in the cell culture. (A) LLC-PK cells were transfected with either control or eIF4E siRNA, as described in the Materials and Methods. Reduced eIF4E expression was verified by Western blotting using antibody against eIF4E. (B) PSaV was then infected at an MOI of 0.2 TCID_50_/cell. Cells were harvested at 24 h postinfection, and RNA was extracted for quantitative reverse transcription-PCR analysis specifically targeting the PSaV protease region. Samples were analyzed in triplicate and in at least three independent experiments. Error bars represent standard errors of the means for triplicate samples.

RNA interference was used to deplete eIF4E, and the effect on PSaV replication was examined. The expression of eIF4E was confirmed after double transfection of siRNA against eIF4E (Fig. 8A), showing a significant reduction in eIF4E expression. Subsequently, inoculation of PSaV showed an eIF4E siRNA-mediated inhibition of virus replication. PSaV mRNA levels were significantly reduced in cells transfected with eIF4E siRNA (Fig. 8B).

## DISCUSSION

In the current study, we have demonstrated that the PSaV VPg protein is associated with the eIF4F translation initiation complex, facilitating viral protein synthesis. Our data fit with those from previous studies on other members of the Caliciviridae, indicating that VPg functions as a proteinaceous cap substitute (9, 10, 19). This novel mechanism of protein-primed translation initiation is also found in members of the Potyviridae family of RNA viruses that infect plants (26, 27). Recent studies also indicate that members of the Astroviridae possess a genome-linked VPg protein and the linkage of VPg to the viral RNA is essential for infectivity (28), although whether in this instance VPg contributes to viral translation has yet to be determined. In the case of potyviruses, the interaction of VPg with eIF(iso)4E is a major determinant of host susceptibility, whereby mutations in eIF(iso)4E lead to resistance to infection (29).

Our data indicate that the recruitment of the eIF4F complex to the 5′ end of PSAV RNA occurs via a direct VPg-eIF4E interaction and that this interaction occurs independently of the cap binding site on eIF4E. In contrast, the interaction of potyvirus VPg with eIF4E competes for cap binding and can inhibit host cell translation (30). Within the Caliciviridae family, the interaction of VPg with eIF4E appears to be conserved, as similar VPg-eIF4E interactions have been described in FCV, a member of the Vesivirus genus, and MNV, a member of the Norovirus genus (9, 10). Importantly, while the interaction of the FCV VPg protein with eIF4E is essential for viral translation, the MNV VPg-eIF4E interaction does not appear to contribute to viral protein synthesis and may play a secondary role in the modulation of eIF4E activity. In the case of MNV, we have recently identified a direct VPg-eIF4G interaction that is essential for MNV translation initiation (31). Our data would indicate that PSaV VPg-dependent translation is functionally identical to that of FCV, in that a VPg-eIF4E interaction is essential, as is the interaction of eIF4E with eIF4G. The functional requirements and possible interaction of VPg with other components of the eIF4F complex are the focus of ongoing studies.

With the data presented in the current study, functional information on the relative requirements of some of the eIF4F components for three of the five genera of the Caliciviridae family, namely, Vesivirus, Norovirus, and Sapovirus, has now been described. However, further studies will be required to unravel the mechanism of translation used by members of the Lagovirus and Nebovirus genera. In the case of lagoviruses, reverse genetics studies would indicate that uncapped *in vitro*-transcribed RNA is infectious when transfected into immortalized cells, indicating that VPg is not essential for the initiation of infection (32). Therefore, while the interaction of translation initiation factors with the VPg proteins of caliciviruses is a conserved feature, subtle differences in the functional requirements of initiation factors between genera are apparent. Similar observations have been made in the study of IRES-mediated translation of the Picornaviridae; for example, the hepatitis A virus IRES requires eIF4E and an intact eIF4G, whereas that of poliovirus and FMDV does not require eIF4E and can function using a cleaved form of eIF4G (33, 34). The biological relevance of the subtle differences in initiation factor requirements for calicivirus translation has yet to be determined; but the differences may in some way have occurred as a consequence of the variation in pathogenesis; i.e., FCV and PSaV typically cause acute self-limiting infections in the natural host, whereas MNV usually causes long-term persistent infection. These differences in the nature of the disease may be at least partially a consequence of differences in the translation efficiency of viral RNA in cells, although further comparative studies on the efficiency of translation are required. Fitting with this hypothesis, the PSaV VPg protein shares a higher degree of sequence similarity with FCV than MNV.

In summary, our study provides additional insights into the novel mechanism of protein-primed VPg-dependent translation initiation used by members of the Caliciviridae family of small positive-sense RNA viruses. This work also highlights how viruses have often evolved novel mechanisms to translate their mRNAs in the presence of high levels of competing cellular mRNAs.
